# Analysis of neonatal hearing screening coverage by State in Brazil

**DOI:** 10.1016/j.bjorl.2026.101839

**Published:** 2026-06-16

**Authors:** Gabriel Caetano de Jesus, Victor Ary Câmara, Arthur Menino Castilho

**Affiliations:** Universidade Estadual de Campinas (UNICAMP), Department of Otorhinolaryngology and Head and Neck Surgery, Campinas, SP, Brazil

**Keywords:** Auditory, Evoked potentials, National health programs, Neonatal screening

## Abstract

**Objectives:**

Analyze the evolution of Neonatal Hearing Screening coverage in Brazil, by region and state, over the 12-years following the publication of the Neonatal Hearing Screening Care Guidelines in 2012, aiming to identify trends and the current situation.

**Methods:**

The study was based on national data from public health care system notifications collected between 2012 and 2023. Segmental regression models were applied to identify changes in temporal trends.

**Results:**

Neonatal Hearing Screening coverage increased from 25.93% in 2012 to 34.43% in 2018 but dropped to 26.39% in 2023. Paradoxically proportion of screenings conducted with Brainstem Auditory Evoked Potential doubled from 2012 to 2023. The South region had the highest average coverage (62.52%), while the North region showed the lowest performance (11.91%). The highest rate in 2023 were 83.19% in the Federal District. These results are limited by the secondary data source from public health and may be affected by underreporting and exclusion of private sector.

**Conclusion:**

Neonatal Hearing Screening coverage in Brazil remains critically unsatisfactory and falls short of the 95% target. The significant decline after 2018 calls for detailed investigation and urgent intervention to reverse this trend.

**Level of evidence:**

4 - Cross-sectional study.

## Highlights

• Neonatal Hearing Screening coverage in Brazil fell to 26.39% in 2023.
• South led with 62.52% and the Federal District reached 83.19% coverage in 2023.
• Auditory Brainstem Response tests have risen in number and proportion since 2018.

## Introduction

The World Health Organization (WHO) has estimated that more than 400 million people have hearing impairments requiring intervention, and this number could exceed 700 million by 2050, representing a morbidity with significant global and economic impact, as the cost of untreated hearing loss exceeds 980 billion dollars annually.^1^ When this impairment is congenital or develops in the early years of life, it affects social and language development. However, when appropriately addressed early on, affected children can reach the developmental levels of their peers by the age of five.^2^

These impacts justify the need for strategies for the prevention, early diagnosis, and timely intervention of hearing loss. In Brazil, this is reflected in Federal Law nº 12.303, from August 2, 2010,^3^ which mandates the performance of hearing screening tests in all maternity hospitals nationwide. A specific protocol was established for the Neonatal Hearing Screening (NHS) strategy through the Neonatal Hearing Screening Care Guidelines (DATAN) in 2012.^4^ This protocol expects Evoked Otoacoustic Emissions (EOA) screening for newborns without risk indicators and Automated (or Screening Mode) Auditory Brainstem Response (ABR) for those with risk indicators. These tests should be performed within the first month of life, ideally within the first 48 h, targeting national coverage rate above 95% as a quality indicator.^4^^,^^5^

However, achieving such neonatal hearing screening coverage levels is a global challenge. Only 21% of countries have coverage rates above 85%, while Brazil is among the majority where national coverage is below the target.^6^

This study aims to assess the evolution of NHS coverage throughout the whole interval since its implementation in Brazil, a 12-years analysis reflecting the consolidation of the national guideline, allowing the evaluation of medium-term terms trends and identification of inflections in coverage such as the impacts of the COVID-19 pandemic.

## Methods

The study was conducted through the collection of public-domain data without individual identification of human subjects and was therefore exempt from approval by the Research Ethics Committee. The official national-level government data were extracted from the DATASUS platform using the online Tabnet tool, available at https://datasus.saude.gov.br/informacoes-de-saude-tabnet/. Data were sourced from the following systems: the Outpatient Information System (SIA-SUS), the Live Birth Information System (SINASC), and the National Supplementary Health Agency (ANS).

The total number of screenings performed within the Brazilian healthcare system ‒ Sistema Único de Saúde (SUS) ‒ was collected by selecting procedures coded as “0211070149 Evoked Otoacoustic Emissions for Hearing Screening (Ear Test)” and “0211070270 Auditory Evoked Potential for Hearing Screening (Ear Test)”, classified by the approved quantity per Region/Federative Unit and year of processing. The number of newborns was collected and classified by Region/Federative Unit and year of processing. As this study assesses only SUS data it presents a limitation from the absent data from private network screening coverage and would underestimate the coverage, but this was partially mitigated by removing from the calculation the number of newborns (< 1-years) covered by supplementary health services in each year from National Supplementary Health Agency (ANS) data.

All data were tabulated using Microsoft Excel, from January 2012 to December 2023. To calculate the Neonatal Hearing Screening (NHS) coverage rate, the following formula was applied: NHS Coverage Rate (%NHS) = [(number of EOA code 0211070149) + (number of ABR code 0211070270)] ÷ [(number of live births) - (population < 1-year covered by supplementary health services)]. This calculation was performed for each Region/Federative Unit and by year. To calculate the proportion of screenings performed using ABR, the following formula was used: ABR Proportion (%ABR) = (number of ABR) ÷ [(number of EOA) + (number of ABR)]. This calculation was also performed for each Region/Federative Unit and by year.

To describe the sample profile according to the variables under study, descriptive statistics were calculated for each year, including mean values and confidence intervals. To evaluate the evolution of the rates over the analyzed period, states without coverage report in any year were excluded and then segmented regression models by joinpoint regression were applied to show trends as the rate of change is not constant and evidence inflection points, using the Joinpoint Regression Program, Version 4.9.1.0., Statistical Research and Applications Branch, National Cancer Institute (2022). This modeling allows us to identify inflection points or change points ‒ where the slope shifts significantly ‒ with statistical significance, enabling a better description of trends in the studied rates by detecting local changes and significant increases or decreases over time.^7^ The significance level adopted for this study was 5% (p < 0.05).

## Results

**Fig. 1:**
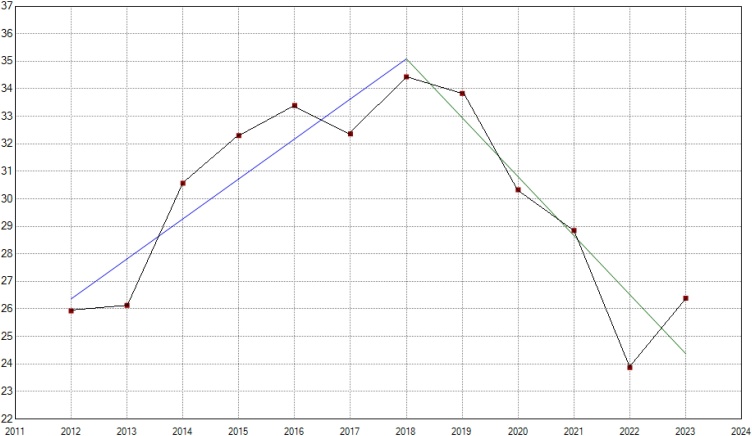
Neonatal Hearing Screening rate (%NHS) in Brazil by year and join point regression. Caption: Neonatal hearing screening coverage in percentage by year between 2012 and 2023. Blue slope = positive 1.45 (p = 0.0110); Green slope = negative 2.14 (p = 0.0065).

The NHS coverage rate in Brazil showed a significant increase from 2012 to 2018 (p-value = 0.0110) with coverage rising from 25.93% to 34.43%, representing a 34% increase over six years. However, from 2018 onward, the trend reversed, showing a significant decline (p-value = 0.0065), reaching only 26.39% in 2023 (Fig. 1).

**Fig. 2:**
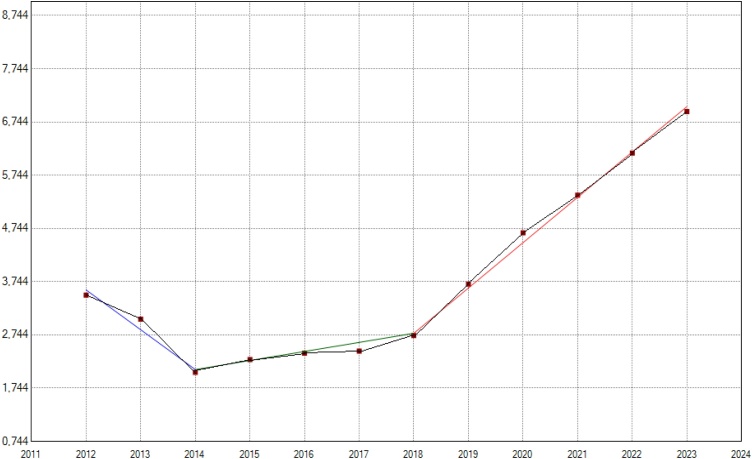
Proportion of Auditory Brainstem Response (%ABR) in Neonatal Hearing Screening by year and join point regression. Caption: Auditory Brainstem Response rate in percentage by year between 2012 and 2023. Blue slope = negative 0.75 (p = 0.0001); Green slope = positive 0.17 (p = 0.0170); Red slope = positive 0.85 (p < 0.0001).

The %ABR initially showed a significant decline from 2012 to 2014 (p-value = 0.0001). However, from 2014 to 2018, the trend reversed, indicating a significant increase (p-value = 0.0170). This upward trend became even more pronounced from 2018 to 2023 (p-value < 0.0001), resulting in a doubling over 12-years (Fig. 2).

**Fig. 3:**
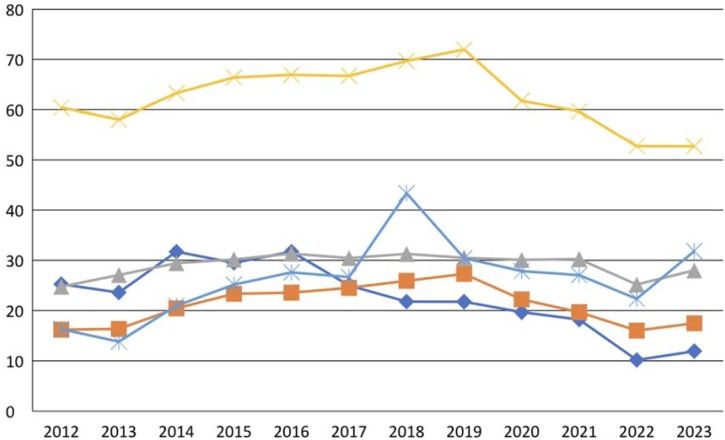
Neonatal Hearing Screening coverage (%NHS) in Brazil by region and year. Caption: Neonatal hearing screening in percentage by region year between 2012 and 2023. Colors: South = yellow; Southeast = gray; Central-west = light-blue; North = dark-blue; Northeast = orange.

The analysis by the five official geographic regions found the highest %NHS in the South region, averaging 62.52% and reaching a peak of 71.97% in 2019, with the current coverage at 52.72% in 2023. Following this was the Southeast region, with an average of 29.04% and a current coverage of 28.01%. The Central-West demonstrated an average of 26.11% and a current 31.82%, while the North registered an average of 22.52% and a current 11.91%. The Northeast region exhibited the lowest coverage, with an average of 21.08% and a current 17.47% (Fig. 3).

**Fig. 4:**
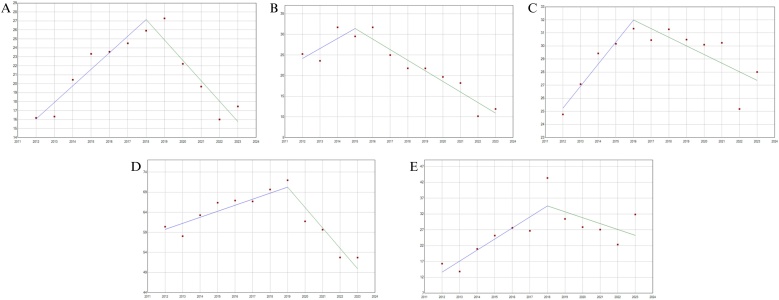
Neonatal Hearing Screening coverage (%NHS) in Brazil by region and year and join point regression. Caption: Neonatal hearing screening in percentage by year in five regions between 2012 and 2023. (A) Northeast, positive slope 1.85*, negative slope 2.28*; (B) North, positive slope 2.42, negative slope 2.56*; (C) Southeast, positive slope 1.69*, negative slope 0.66*; (D) South, positive slope 1.50*, negative slope 5.08*; (E) Central-west, positive slope 3.49*, negative slope 1.86. * Indicates p < 0.05.

The segmented regression by geographic region showed a similar pattern with the national one, with a significant increase in the early years, followed by a significant decline in the later years (Fig. 4).

**Fig. 5:**
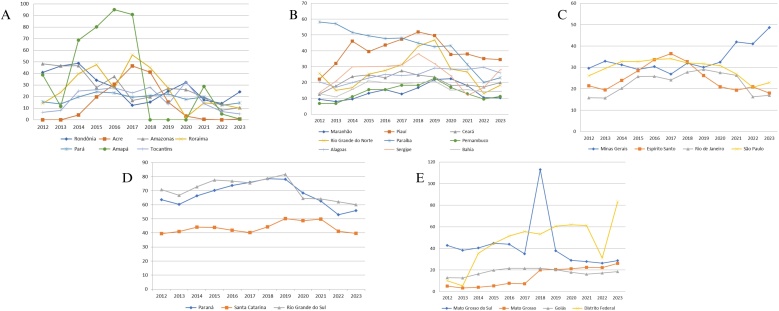
Neonatal Hearing Screening coverage (%NHS) in Brazil by states from the five regions and year. Caption: Neonatal hearing screening in percentage by year in 26 States and federal district between 2012 and 2023. (A) North; (B) Northeast; (C) Southeast; (D) South; (E) Central-west.

The %NHS by state in each geographic region shows that the states with the highest average coverage were Rio Grande do Sul (70.98%), Paraná (67.24%), and the Federal District (46.14%). The highest current %NHS rates in 2023 were 83.19% in the Federal District, 60.07% in Rio Grande do Sul, and 55.89% in Paraná (Fig. 5). Throughout the study period one single year had a coverage rate over 100%, observed in Mato Grosso do Sul in 2018 (112.99%). The lowest average coverage rates were observed in Acre (13.48%), Mato Grosso (13.78%), and Pernambuco (13.79%) (Fig. 5). Two states had years without reported screening, Acre with no reports in 2012 and 2022, and Amapá with no reports in 2018 and 2019.

**Fig. 6:**
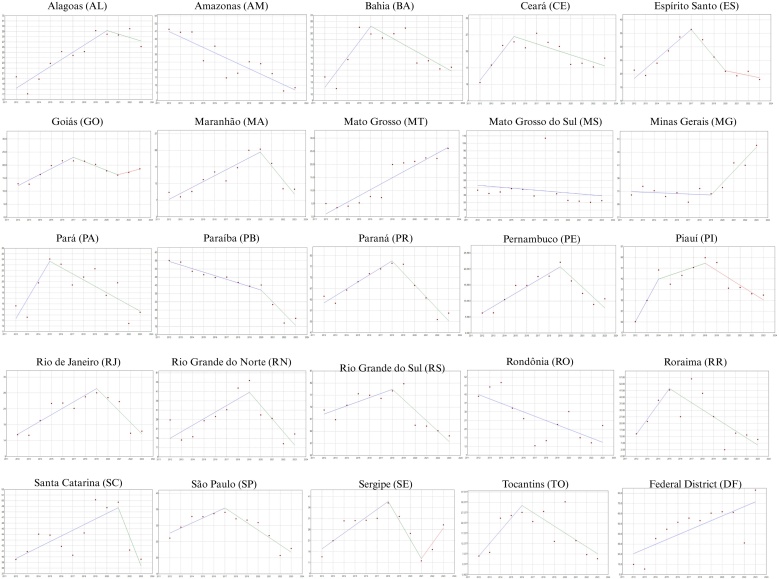
Neonatal Hearing Screening coverage (%NHS) in Brazilian states and the Federal District join point regression. Caption: Neonatal hearing screening in percentage by year in 24 states and federal district between 2012 and 2023 join point regression and its slope values: Alagoas = +1.37*, −0.63; Amazonas = −3.45*; Bahia = +2.51*, −1.05*; Ceará = +4.42*, −1.11*; Espírito Santo = +3.62*, −5.08. −0.87; Goiás = +2.18*, −1.68. −1.09; Maranhão = +1.78*, −4.21*; Mato Grosso = +2.31*; Mato Grosso do Sul = −1.29; Minas Gerais = −0.18, +4.58*; Pará = +3.44; −1.12*; Paraíba = −2.18*; −7.17*; Paraná = +3.18*; −5.45*; Pernambuco = +2.06*; −3.21*; Piauí = +9.90, +1.83, −3.40*; Rio de Janeiro = +2.06*; −3.57*; Rio Grande do Norte = +3.52*; −7.06*; Rio Grande do Sul = +1.71, −4.34*; Rondônia = −2.50*; Roraima = +11.61, −5.38*; Santa Catarina = +1.01*, −5.18*; São Paulo = +1.56*, −2.29*; Sergipe = +3.59*, −8.65, +6.94; Tocantins = +6.11, −3.38*; Federal District = +4.64*. * Indicates p < 0.05.

The joint regression analysis by state, excluding states with periods of no reported data, find two-thirds of the states exhibited a biphasic pattern similar to the national analysis, with positive growth in the early years followed by a negative inflection, most frequently occurring in 2018 and 2019. Rondônia, Amazonas, Paraíba, and Mato Grosso do Sul showed a consistently negative trend throughout the entire period, while Mato Grosso and the Federal District exhibited a continuous positive trend. These trends are collectively shown in Fig. 6, and individual states graphics can be found in complementary material.

## Discussion

Twelve years after the publication of DATAN, neonatal hearing screening coverage in Brazil stands at only 26.39%, still far from the universal coverage recommended by the guideline.^4^ This goal was reinforced at the latest Joint Committee on Infant Hearing in 2019 at a global level, which establishes that screening should be completed within the first month of life and ideally before hospital discharge.^8^

The current coverage is less than 1% higher than the rate observed in 2012, which contradicts the expectations set by previous national studies with data up to 2018 that identified a growing trend until then.^9^, ^10^, ^11^ Though the model in this present study demonstrates, through segmented regression, that the %NHS trend in Brazil showed significant growth until 2018, it shifted to a significant decline that has persisted since then (Fig. 1). This pattern is consistent across the country’s geographic regions (Fig. 4, Fig. 5), suggesting that events with significant impact from that year onward contributed to the downward trend across regions.

Simultaneously Brazil's Human Development Index (HDI) ‒ which reflects a country's income, education, and healthcare quality ‒ declined from 0.764 in 2019 to 0.758 in 2020 and 0.756 in 2021. This trend aligns with international reports showing a global decline, largely associated with the COVID-19 pandemic, which began in 2020.^12^ This raises the hypothesis that the pandemic's impact may have contributed to the decline in %NHS, possibly due to reduced access to healthcare. However, studies from other countries have not found an association between the pandemic and a decline in screening coverage, although they did observe an increase in loss to follow-up after screening failure.^13^, ^14^, ^15^ Even in Brazil, the technical note from the Multiprofessional Committee on Hearing Health (COMUSA) in 2020 recommended that, despite the pandemic, the first screening should still be performed before hospital discharge, following the recommended Personal Protective Equipment (PPE) guidelines, which should not have led to a decrease in NHS coverage.^16^ The study presents a limitation from its secondary data potential underreporting of procedures in SIA-SUS by healthcare professionals and should be considered as a major factor for this low coverage, but nonetheless alarms for other hypotheses not addressed in this study, which should be further investigated using primary data in new studies as availability of trained professionals as speech therapists and lack of properly maintained or replaced equipment.

Although insufficient to determine another significant positive inflection, it is noteworthy that the last year %NHS was higher than the previous, both at the national average and across geographic regions, as well as in 70% of states. This could mark the beginning of a new upward trend, which could be further studied. Additionally, the recent increase in coverage observed in the Federal District, Mato Grosso, and Minas Gerais, contrary to the overall decline in the country, should be further investigated ‒ particularly the Federal District, which came closest to the target universal coverage.

The percentage of screenings performed with ABR among all screenings each year (%ABR) showed a slight upward trend between 2014 and 2018, followed by a significant and even more pronounced increase since 2018, occurring parallel to the decline in %NHS. This indicates not only a relative increase but also a rise in the absolute number of ABR tests conducted in recent years, which goes in the opposite direction of %NHS decline, weakening the hypothesis of observed trends been merely due to underreporting issues.

The ABR test is performed instead of the OAE as a screening method for newborns with risk indicators, according to DATAN.^4^ There is a possibility of an increase in births with risk factors during the period, however, caution is needed when attempting to link this with to the COVID-19 pandemic, as maternal infection with the virus has not been clearly shown to be a risk factor for congenital hearing loss.^17^^,^^18^

As expected, considering the regional particularities of a country with a continental size, it was found some inconsistencies in data reporting from some states ‒ particularly those that failed to report data in certain years. A notable example is Amapá, which had a screening coverage up to 95.01% in 2016 but 0% in 2018 and 2019. Such inconsistencies suggest a bias due to incomplete data reporting failures. Another atypical finding was the 112.99% screening coverage in Mato Grosso do Sul in 2018. This can be understood based on the calculation method used in this study, which relies on aggregated data and an estimate of the exposed population. Possible explanations for this value include migration to another healthcare region in larger centers, retests being reported with test code leading to duplicate records for the same patient, delayed notifications causing an accumulation of retroactive records, overlap with the population excluded due to private healthcare coverage, among others.

This study was derived from DATASUS, which contains official government-approved whole data at the national level. Even considering the main limitations of under notification and the impact of outsourcing that occurs in SUS services on the DATASUS notification, and also the impact of the incomplete data from private network, only partially suppressed, or regional inconsistency bias, it still holds significant relevance as it is a review of the largest available database to date, serving as a source for public health analyses searching causality for found trends.

## Conclusion

The neonatal hearing screening coverage rate showed a significant decline starting in 2018, with consistency among states, reaching only 26.39% in 2023, which is alarming, and even the unit with the highest coverage in 2023, Federal District at 83.19%, remain below the 95% target established over 12-years ago in the guidelines. Paradoxically, the decline in the number of OAEs has been accompanied by an increase in the number of ABR tests since 2018, drawing attention to the investigation of newborns with risk indicators. The understanding of the particularities causing these trends should be assessed to enable decisive intervention in public health to reverse it, as failure to address this issue results in high annual costs. It is imperative to aim for the universal coverage target, highlighting the need to revisit the execution strategy of DATAN.

## ORCID ID

Gabriel Caetano de Jesus: 0000-0003-4217-0553

Victor Ary Câmara: 0000-0003-0861-5782

Arthur Menino Castilho: 0000-0002-9024-8004

## Data availability

The authors declare that all data are available in repository.

## Declaration of competing interest

The authors declare no conflicts of interest.
